# Viral etiologies of influenza‐like illness and severe acute respiratory infections in Thailand

**DOI:** 10.1111/irv.12554

**Published:** 2018-05-08

**Authors:** Malinee Chittaganpitch, Sunthareeya Waicharoen, Thitipong Yingyong, Prabda Praphasiri, Somchai Sangkitporn, Sonja J. Olsen, Kim A. Lindblade

**Affiliations:** ^1^ National Institute of Health Ministry of Public Health Nonthaburi Thailand; ^2^ Bureau of Epidemiology Ministry of Public Health Nonthaburi Thailand; ^3^ Influenza Program Thailand Ministry of Public Health ‐ U.S. Centers for Disease Control and Prevention Collaboration Nonthaburi Thailand; ^4^ Influenza Division Centers for Disease Control and Prevention Atlanta GA USA

**Keywords:** influenza‐like illness, realtime PCR, respiratory viruses, severe acute respiratory infections

## Abstract

**Background:**

Information on the burden, characteristics and seasonality of non‐influenza respiratory viruses is limited in tropical countries.

**Objectives:**

Describe the epidemiology of selected non‐influenza respiratory viruses in Thailand between June 2010 and May 2014 using a sentinel surveillance platform established for influenza.

**Methods:**

Patients with influenza‐like illness (ILI; history of fever or documented temperature ≥38°C, cough, not requiring hospitalization) or severe acute respiratory infection (SARI; history of fever or documented temperature ≥38°C, cough, onset <10 days, requiring hospitalization) were enrolled from 10 sites. Throat swabs were tested for influenza viruses, respiratory syncytial virus (RSV), metapneumovirus (MPV), parainfluenza viruses (PIV) 1‐3, and adenoviruses by polymerase chain reaction (PCR) or real‐time reverse transcriptase‐PCR.

**Results:**

We screened 15 369 persons with acute respiratory infections and enrolled 8106 cases of ILI (5069 cases <15 years old) and 1754 cases of SARI (1404 cases <15 years old). Among ILI cases <15 years old, influenza viruses (1173, 23%), RSV (447, 9%), and adenoviruses (430, 8%) were the most frequently identified respiratory viruses tested, while for SARI cases <15 years old, RSV (196, 14%) influenza (157, 11%) and adenoviruses (90, 6%) were the most common. The RSV season significantly overlapped the larger influenza season from July to November in Thailand.

**Conclusions:**

The global expansion of influenza sentinel surveillance provides an opportunity to gather information on the characteristics of cases positive for non‐influenza respiratory viruses, particularly seasonality, although adjustments to case definitions may be required.

## 1 INTRODUCTION

The establishment of influenza viral surveillance systems has become the cornerstone of preparations for the next influenza pandemic.^1^, ^2^ The number of countries conducting surveillance for seasonal influenza viruses has increased substantially in recent years, leading to a better understanding of the epidemiology of influenza, particularly in tropical countries where influenza virus surveillance had been limited before avian influenza reemerged in the late 1990s.^3^ However, generally only 6%‐21% of respiratory infections are associated with influenza viruses in the tropics,^4^, ^5^, ^6^, ^7^ leaving a significant number of infections for which the etiology is unknown.

With increasing recognition of the importance of non‐influenza infections as causes of acute respiratory illnesses, many countries have added diagnostics for coronaviruses, respiratory syncytial virus (RSV), adenovirus, parainfluenza (PIV) viruses 1‐4, metapneumovirus (MPV), and rhinoviruses to influenza surveillance platforms.^3^ While case definitions and other aspects of influenza virus surveillance may not be optimally suited for other respiratory viruses, these systems have contributed to a better understanding of the global burden of respiratory viruses in general. However, information on non‐influenza respiratory viruses is still lacking in many tropical countries.

The National Institute of Health in Thailand (NIH) at the Ministry of Public Health (MOPH), in collaboration with the US Centers for Disease Control and Prevention (CDC), established sentinel surveillance for ILI in 2004 in 11 sites distributed throughout the country; SARI surveillance was initiated in 5 sites between 2009 and 2011, although the overall number of sites had decreased to 9 by 2012.^8^ The surveillance system was established to monitor the frequency of influenza virus infection, identify new strains, and describe seasonality. In 2008, testing for 6 additional respiratory viruses was initiated. We present results of this expanded testing and compare characteristics of cases with different respiratory viral infections.

## 2 METHODS

### 2.1 Surveillance sites, case enrollment, and sample collection

Thailand is an upper middle‐income country located in Southeast Asia north of the Equator and South of the Tropic of Cancer. Annual mean temperature is 27.5°C, and annual rainfall total is approximately 1700 mm, with August and September recording the most rainfall.^9^

**Figure 1:**
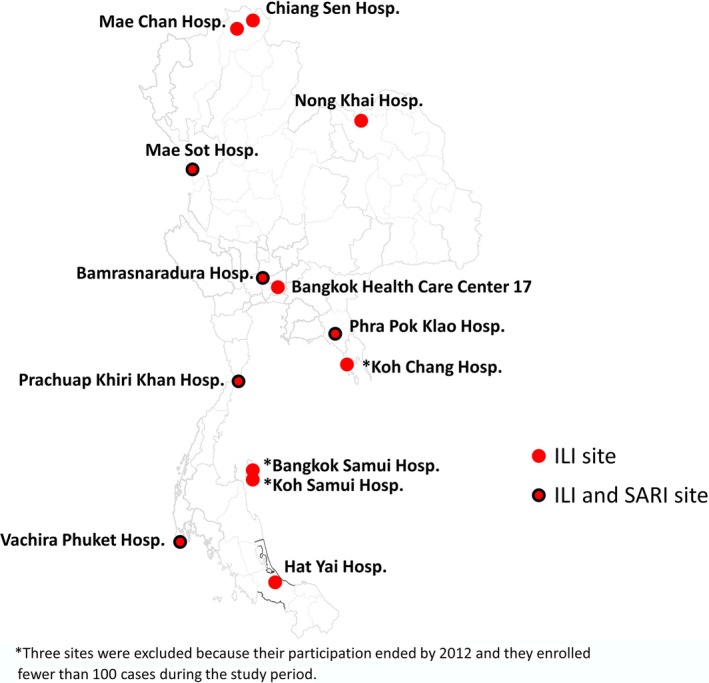
Map of influenza surveillance sites, Thailand, 2010‐2014

Sentinel surveillance for influenza viruses was established in Thailand in 2004 as routine public health surveillance and has been described in more detail elsewhere.^8^ Hospitals were selected to represent major urban areas in all regions of Thailand. Enrollment for influenza‐like illness (ILI) began in 2004 while enrollment for severe acute respiratory infection (SARI) began in 2010 in certain hospitals, selected because of their capacity and willingness to enroll additional cases. To enable contemporaneous comparisons of ILI and SARI cases, and cover the entire influenza season in Thailand, we have restricted data to the period June 2010 through May 2014. During this period, cases were enrolled from 13 hospitals, but 3 were excluded from analysis because their participation ended by 2012, and they each enrolled fewer than 100 cases between 2010 and 2012 (Figure 1). ILI was modified from the WHO case definition as documented fever (axillary temperature ≥ 38°C) or history of fever and cough and illness not requiring hospitalization.^10^ SARI was defined as documented or history of fever and cough, onset within 10 days of presentation, and severity of illness requiring hospitalization. Each site was instructed to enroll up to 10 case‐patients per week (5 persons <15 years and 5 persons ≥15 years).

### 2.2 Sample collection and laboratory testing

Throat swab specimens were collected from enrolled patients and stored in viral transport media. The vials were kept at 2‐4°C for up to 4 hours, then moved to a liquid nitrogen tank at the hospital laboratory and transported weekly to the Thai NIH outside of Bangkok. Specimens were tested for influenza A and B viruses using the standard World Health Organization (WHO) and CDC protocol for real‐time reverse transcription‐polymerase chain reaction (rRT‐PCR).^11^

Samples were tested using PCR for adenoviruses and rRT‐PCR for RSV, MPV, and PIV 1‐3 using methods described for individual rRT‐PCR assays in Kodani et al.^12^ Mixed infections were classified in order of the most frequently identified virus families first, that is, any virus found in a specimen with influenza was classified as an influenza mixed infection, followed by RSV, adenovirus, MPV, PIV‐1, PIV‐3, and PIV‐2.

### 2.3 Data analysis

We explored differences in characteristics of cases of ILI and SARI, including the prevalence of specific viral respiratory pathogens, by age (<15 years and ≥15 years) using the chi‐square test. Differences in the median number of days cases were ill before presenting to the health facility were compared using the Mann‐Whitney *U* test. Percentages are proportions of non‐missing data. The proportion of specimens found positive for respiratory viruses tested each month was plotted over time to describe annual and seasonal patterns. The mean monthly proportions positive for respiratory viruses were calculated for each calendar month to provide a summary of the seasonal patterns. All analyses were conducted using sas (Statistical Analysis Software 9.3, Cary, NC, USA).

### 2.4 Human subjects

Data were collected as part of routine public health surveillance, and written consent was not required.

## 3 RESULTS

We screened 15 369 persons between June 1, 2010 and May 31, 2014, of whom 12 951 (84%) were outpatients, 2378 (16%) were inpatients, and 40 (0.3%) did not have admission status recorded. We identified 8106 (53%) cases of ILI and 1754 (11%) cases of SARI; 5509 (36%) patients did not meet either case definition. Among the 4715 outpatients who did not meet an ILI case definition, 2484 (53%) did not have a documented or history of fever, and 2541 (54%) did not present with a cough. Among the 754 inpatients who did not meet a SARI case definition, 394 (52%) did not have a documented or history of fever, 294 (39%) did not have a cough, and 145 (19%) had an illness duration that was unknown or >10 days.

There were 5069 (63%) ILI patients and 1404 (80%) SARI patients <15 years old. SARI patients were more likely to be infants or older adults than ILI patients. Among ILI patients <15 years old, the median age was 3 years (interquartile range [IQR] 1‐7), while the median age for SARI patients <15 years old was 2 years (IQR1‐4). Among patients ≥15 years old, the median age was 34 (IQR 23‐49) and 59 years (IQR 40‐74), for ILI and SARI, respectively. Among persons <15 years old, a greater proportion of SARI cases was <1 year old (341, 24%) than ILI cases (658, 13%, *P *<* *.001) (Table 1). In comparison, among patients ≥15 years old, a greater proportion of SARI cases (141, 40%) was ≥65 years old compared with ILI patients (274, 9%; *P *<* *.001).

**Table 1:** Frequency of demographic and clinical characteristics of cases of influenza‐like illness (ILI) and severe acute respiratory infection (SARI) by age category, Thailand, 2010‐2014^a^

Characteristics	ILI			SARI		
	<15 years (N = 5069)	≥15 years (N = 3037)	*P* *	<15 years (N = 1404)	≥15 years (N = 350)	*P* *
	n (%)	n (%)		n (%)	n (%)	
Age (years)						
<1	658 (13%)			341 (24%)		
≥65		274 (9%)			141 (40%)	
Sex, male	2692 (53%)	1484 (49%)	.0003	828 (59%)	172 (49%)	.001
Days ill, median (IQR)	2 (1‐3)	2 (1‐3)	.51	2 (1‐3)	2 (1‐3)	.72
Symptoms						
Cough^b^	5069 (100)	3037 (100)		1404 (100)	350 (100)	
History of fever	5054 (100)	3011 (99)	.0006	1397 (100)	349 (100)	.59
Documented fever ≥ 38°C	3867 (84)	2454 (88)	<.0001	1227 (91)	273 (79)	<.0001
Rhinorrhea	4158 (82)	2134 (70)	<.0001	1169 (83)	271 (77)	.01
Headache	1631 (32)	2106 (69)	<.0001	193 (14)	154 (44)	<.0001
Sore throat	2020 (40)	2100 (69)	<.0001	288 (21)	104 (30)	.0002
Muscle aches	577 (11)	1888 (62)	<.0001	71(5)	110 (31)	<.0001
Fatigue	1084 (21)	1078 (36)	<.0001	159 (11)	154 (44)	<.0001
Vomiting	1690 (33)	440 (14)	<.0001	473 (34)	38 (11)	<.0001
Nausea	955 (19)	551 (18)	.43	153 (11)	37 (11)	.86
Shortness of breath	690 (14)	315 (10)	<.0001	389 (28)	205 (59)	<.0001
Diarrhea	441 (9)	149 (5)	<.0001	197 (14)	20 (6)	<.0001

^*^The *P* value was calculated using the chi‐square distribution to compare the characteristics and symptoms of cases within each case definition by age category, except for the differences in median number of days between illness onset and presentation to the health facility which were compared using the Mann‐Whitney *U* test.

^a^Percentages are a proportion of non‐missing data. ILI, influenza‐like illness; SARI, severe acute respiratory infection; IQR, interquartile range.

^b^The chi‐square statistic cannot be calculated because at least one cell has 0 cases.

The median number of days between symptom onset and presentation at the surveillance hospital (days ill) was 2 (IQR 1‐3), and there was no difference by case definition or age category (Table 1). Cough and history of fever were present in all (100%) cases. Documented fever was found in 3867 (84%) ILI and 1227 (91%) SARI patients <15 years old, and in 2199 (90%) ILI and 273 (79%) SARI patients ≥15 years. Among other symptoms, rhinorrhea was the most common for both ILI and SARI, reported by 3598 (81%) and 1844 (69%) of ILI patients <15 and ≥15 years, respectively, and by 1169 (83%) and 271 (77%) SARI patients <15 and ≥156 years, respectively.

### 3.1 Identification of respiratory virus infections by case definition and age category

**Figure 2:**
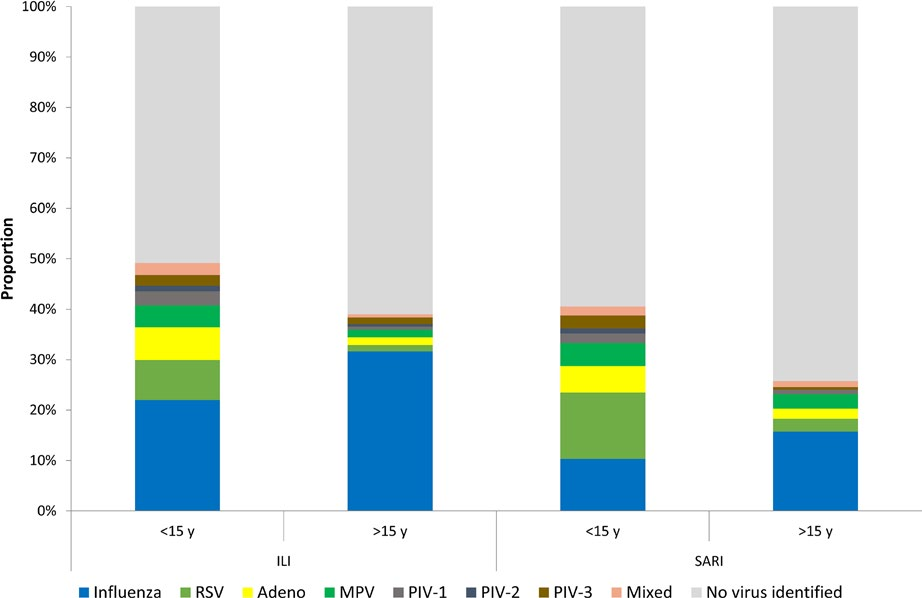
Distribution of respiratory viruses identified in cases of influenza‐like illness and severe acute respiratory infection by age category, Thailand, 2010‐2014

The proportion of cases with any respiratory virus among those tested identified ranged from 26% (among SARI patients ≥15 years old) to 49% (among ILI cases <15 years old) (Table 2 and Figure 2). Influenza viruses were detected in 1173 (23%) and 975 (32%) ILI patients <15 and ≥15 years old, respectively, and in 157 (11%) and 58 (17%) SARI cases <15 and ≥15 years, respectively. RSV was identified in 447 (9%) ILI cases and in 196 (14%) SARI cases <15 years old, but was identified in few cases of either ILI or SARI in persons ≥15 years. Adenovirus was found in 430 (8%) of ILI and 90 (6%) of SARI cases <15 years old. Metapneumovirus was found in 245 (5%) ILI cases and 68 (5%) SARI cases <15 years old. The prevalence of other respiratory viruses was ≤3% in any age category or case definition. Mixed virus infections (ie, more than one virus family) were identified in 141 (3%) ILI and 25 (2%) SARI cases <15 years. Among ILI cases <15 years old with mixed infections, adenoviruses were most commonly identified, found in 101 (72%) mixed infections, followed by influenza viruses (77, 55%) and RSV (46, 33%). Among SARI cases <15 years old with mixed infections, adenovirus was the most common virus (16, 64%), with both influenza viruses and RSV found in 12 (48%) mixed infections. A supplementary data file provides the number of tests and positive results for each type of respiratory virus by month (Table S1).

**Table 2:** Numbers and percentages for respiratory viruses identified among cases of influenza‐like illness (ILI) and severe acute respiratory infection (SARI) by age category, Thailand, 2010‐2014

Viral respiratory pathogen	ILI			SARI		
	<15 years (N = 5069)	≥15 years (N = 3037)	*P* *	<15 years (N = 1404)	≥15 years (N = 350)	*P* *
	n (%)	n (%)		n (%)	n (%)	
Influenza viruses	1173 (23)	975 (32)	<.0001	157 (11)	58 (17)	.006
Respiratory syncytial virus	447 (9)	40 (1)	<.0001	196 (14)	10 (3)	<.001
Adenoviruses	430 (8)	56 (2)	<.0001	90 (6)	8 (2)	.0026
Metapneumovirus	245 (5)	45 (1)	<.0001	68 (5)	12 (3)	.26
Parainfluenza virus ‐ 1	155 (3)	19 (0.6)	<.0001	27 (2)	3 (0.9)	.17
Parainfluenza virus ‐ 2	59 (1)	18 (0.6)	.01	14 (1)	0 (0)	
Parainfluenza virus ‐ 3	127 (3)	44 (1)	.001	42 (3)	3 (0.9)	.02
Mixed infections	141 (3)	19 (0.6)	<.0001	25 (2)	4 (1)	.40
Any virus infection	2492 (49)	1184 (39)	<.0001	569 (41)	90 (26)	<.0001

ILI, influenza‐like illness; SARI, severe acute respiratory infection.

^*^The *P* value was calculated using the chi‐square distribution to compare the number of cases of different respiratory viruses within each case definition by age category. The *P* value was not calculated if there was a cell with zero cases.

### 3.2 Seasonal trends in respiratory viruses

**Figure 3:**
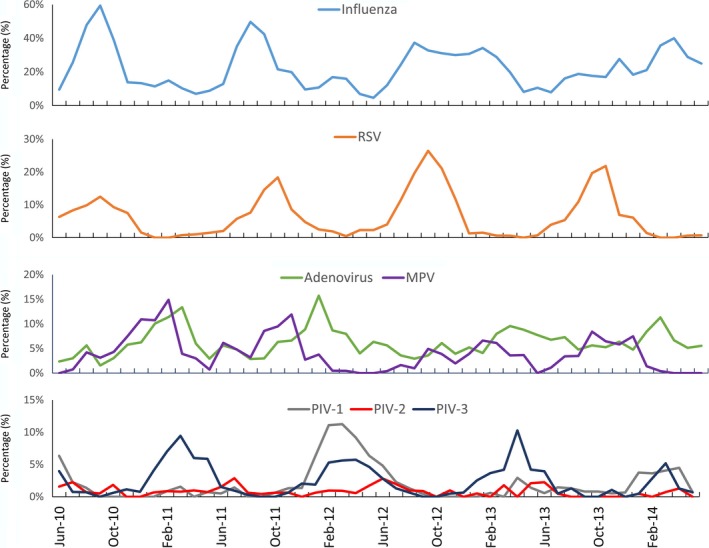
Seasonal trends in respiratory viruses as the proportion of all cases testing positive for specific respiratory viruses, Thailand, 2010‐2014 (Cases include those that meet either the influenza‐like illness or severe acute respiratory infections case definition)

Between 2010 and 2014, the proportion of samples testing positive for influenza viruses never fell below 4% in any month; no other respiratory virus included in the panel demonstrated perennial transmission (Figure 3). Despite year‐round transmission, influenza viruses had 1‐2 seasonal peaks each year: in 2010, 2011, and 2012, the peak for influenza was between August and September, with smaller secondary peaks between January and February. In 2013, however, the pattern changed and the proportion of cases positive for influenza began increasing in July and finally peaked in March 2014. RSV consistently demonstrated a single, sharp peak in either September or October each year between 2010 and 2014. Adenovirus and MPV had sharper peaks in 2011 and 2012 and no clear seasonal pattern through 2013 and 2014. PIV‐1 had one large increase in February and March 2012, while PIV‐3 showed seasonal increases between March and April each year. PIV‐2 showed no particular seasonal pattern.

**Figure 4:**
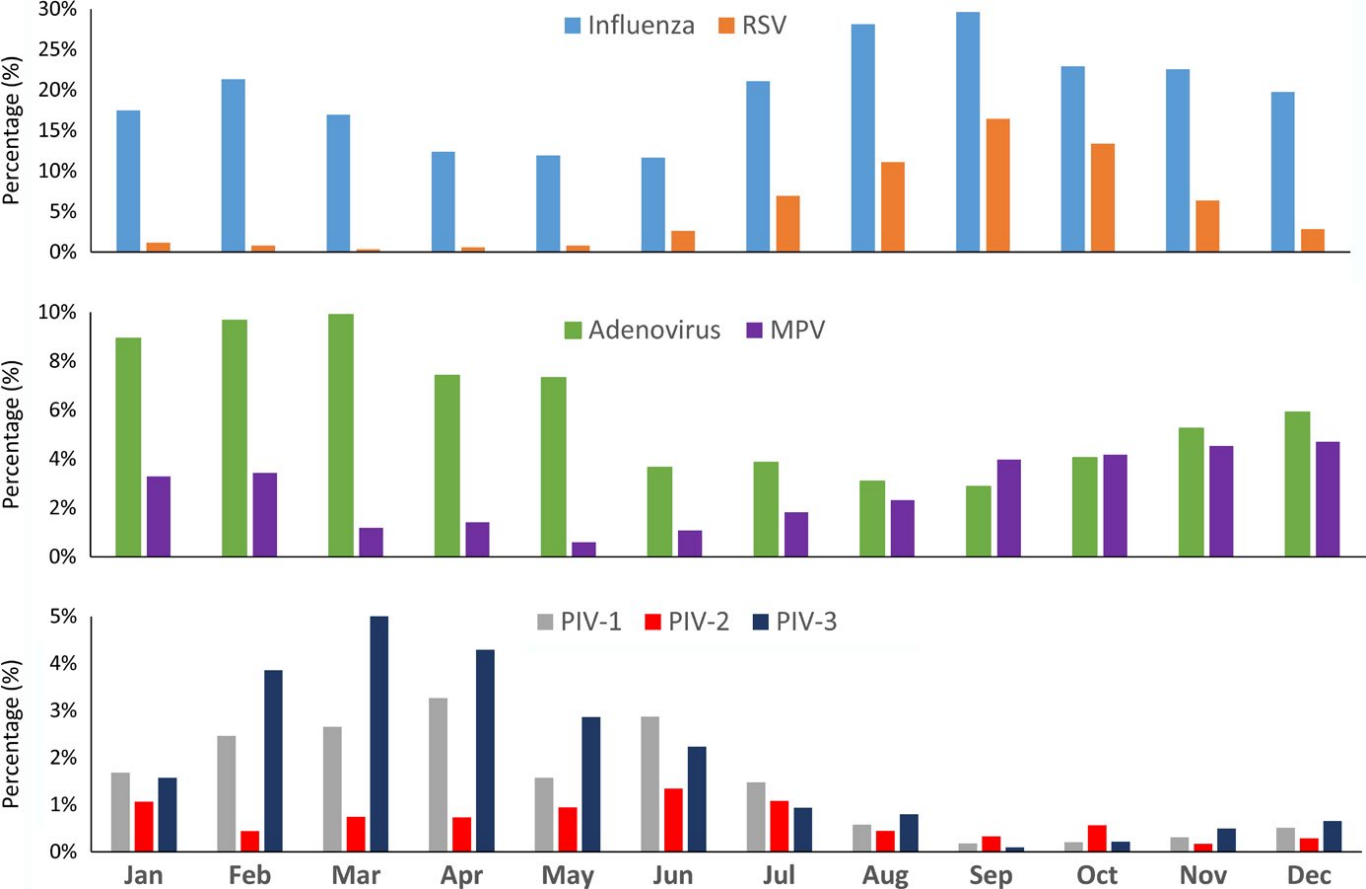
Annual seasonality patterns of respiratory viruses as the mean proportion positive per calendar month, Thailand, 2010‐2014 (Cases include those that meet either the influenza‐like illness or severe acute respiratory infections case definition)

These seasonal patterns were made clearer by averaging the proportion of samples positive for respiratory viruses by calendar month. Influenza viruses had 2 peaks in February and September; these peaks correspond with the Northern Hemisphere and the Southern Hemisphere seasonality, respectively (Figure 4). RSV and MPV experienced their peaks after the rains in September and October. Adenoviruses peaked during the northern hemisphere winter months. PIV‐1 and PIV‐3 showed clear peaks in March and April, while a seasonal pattern for PIV‐2 was not apparent. There were no differences in seasonal patterns by age grouping for any of the viruses (data not shown).

## 4 DISCUSSION

Influenza viruses were the most common respiratory viruses diagnosed among ILI and SARI cases enrolled in a sentinel surveillance system designed to capture influenza‐positive patients in Thailand. RSV, adenoviruses, MPV, and PIV 1‐3 were also identified in ILI and SARI cases. Although the surveillance system was designed to detect influenza viruses, important information on the burden and seasonality of cases of ILI and SARI associated with non‐influenza respiratory viruses can be obtained.

There were differences in the distribution of the respiratory viruses tested by age <15 and ≥15 years. In both ILI and SARI cases, influenza viruses were less likely to be identified in persons <15 years old than they were in persons ≥15 years, whereas RSV was primarily an infection of children, particularly among SARI cases. Similar age‐related patterns have been identified among inpatients in Lao People's Democratic Republic, Guatemala, and Egypt^5^, ^13^, ^14^ and previously in Thailand.^15^ Among ILI cases, the other respiratory viruses (adenovirus, MPV, and PIV 1‐3) were also more likely to be found in children <15 years than in persons ≥15 years. Among SARI cases, the age pattern was similar as for ILI, but the difference in prevalence of infection by age group was only statistically significant for adenoviruses and PIV‐3.

The seasonal pattern for influenza in Thailand demonstrated 2 peaks, one in February and the other in September, but influenza viruses were transmitted perennially. The second influenza peak in September overlapped considerably with the peak in RSV cases. RSV transmission has been associated with the rainy season in many tropical countries,^16^, ^17^, ^18^, ^19^, ^20^ and Thailand has shown a similar pattern.^15^ MPV had a higher peak after the rains, but transmission was also high during the cooler, drier months of January and February. A study of hospitalized patients with lower respiratory tract infections in Thailand found that PIV‐1 and PIV‐3 were detected most frequently from January‐May,^21^ similar to our results. As vaccines for RSV are under development, better information on the seasonality of RSV in different parts of the world will help in determining the timing of future vaccination campaigns.

The ILI and SARI case definitions were developed for influenza surveillance and may not be optimal for the detection of non‐influenza respiratory viruses, including RSV in young infants.^22^, ^23^ An additional potential limitation to the detection of respiratory viruses in this surveillance system was the use of throat rather than nasal or nasopharyngeal swabs. A paired comparison of throat and nasopharyngeal swabs in Kenya demonstrated that among SARI patients, throat swabs were less likely than nasopharyngeal swabs to yield influenza B virus, PIV‐2 and PIV‐3 but more likely to yield adenovirus and influenza A virus.^24^ The Kenya study found no difference in yield of PIV‐1, MPV, and RSV by swab type. Our results, therefore, may have underestimated the amount of influenza B virus, PIV‐2, and PIV‐3 among cases included in the surveillance system. Specimens were not tested for either rhinoviruses or coronaviruses, which are both common causes of acute respiratory infections and would likely have increased the proportion of specimens with any respiratory virus identified.

The generalizability of these results to all of Thailand is dependent on the representativeness of the sites chosen for sentinel surveillance and of the patients who contributed data and specimens. The sites were chosen from the 5 regions of Thailand but were located in large urban centers and may not be representative of more rural settings. Thailand has a universal healthcare coverage scheme that ensures good access to care for a large proportion of the country, which strengthens the generalizability of the results to the general population. However, the sentinel sites did not select a probability sample of patients, and it is possible that there was some unmeasured bias in the convenience sample of patients participating in the surveillance. However, we have no reason to believe that any systematic bias prevents these data from being generalizable to the country as a whole.

As has been found in other tropical countries, ILI and SARI in Thailand can be caused by a variety of respiratory viruses in addition to influenza. Influenza virus surveillance systems can be used to detect and describe the characteristics of other respiratory viruses. However, some adjustments may be required to calculate an accurate burden of non‐influenza respiratory viruses due to differences in their clinical presentation. Given the global expansion of influenza surveillance systems, adapting ILI and SARI case definitions to include non‐influenza respiratory viruses will be helpful in the future to measure the impact of new control measures for non‐influenza respiratory viruses.

## DISCLAIMER

The findings and conclusions in this report are those of the authors and do not necessarily represent official position of the US Centers for Disease Control and Prevention.
